# Pathways in Skeletal Muscle: Protein Signaling and Insulin Sensitivity after Exercise Training and Weight Loss Interventions in Middle-Aged and Older Adults

**DOI:** 10.3390/cells10123490

**Published:** 2021-12-10

**Authors:** Alice S. Ryan, Guoyan Li, Shawna McMillin, Steven J. Prior, Jacob B. Blumenthal, Laura Mastella

**Affiliations:** 1 VA Research Service, VA Maryland Health Care System, 10 N Greene Street, Baltimore, MD 21201, USA; 2 Department of Medicine, Division of Gerontology and Palliative Medicine, University of Maryland School of Medicine, 655 W Baltimore Street, Baltimore, MD 21201, USA; guoyanli@som.umaryland.edu (G.L.); mcmil318@umn.edu (S.M.); Jacob.Blumenthal@va.gov (J.B.B.); 3 Baltimore VA Medical Center Geriatric Research, Education and Clinical Center, 10 N Greene Street, Baltimore, MD 21201, USA; sprior@som.umaryland.edu (S.J.P.); Laura.Mastella@va.gov (L.M.)

**Keywords:** MAPK pathway, AKT pathway, Insulin pathway, hyperinsulinemic-euglycemic clamp, body composition, aerobic exercise training, weight loss, insulin sensitivity, obesity

## Abstract

Aging and obesity contribute to insulin resistance with skeletal muscle being critically important for maintaining whole-body glucose homeostasis. Both exercise and weight loss are lifestyle interventions that can affect glucose metabolism. The purpose of this study was to examine the effects of a six-month trial of aerobic exercise training or weight loss on signaling pathways in skeletal muscle in the basal condition and during hyperinsulinemia during a glucose clamp in middle-aged and older adults. Overweight and obese men and women aged 50–70 years were randomly allocated and completed six months of either weight loss (WL) (*n* = 18) or 3x/week aerobic exercise training (AEX) (*n* = 17). WL resulted in 10% weight loss and AEX increased maximal oxygen consumption (VO_2_max) (both *p* < 0.001). Insulin sensitivity (hyperinsulinemic-euglycemic 80 mU·m^−2^·min^−1^ clamp) increased in WL and AEX (both *p* < 0.01). In vivo insulin stimulation increased phosphorylation/total protein ratio (P/T) of protein kinase B (Akt), glycogen synthase kinase 3 beta (GSK-β3), 70 kDa ribosomal protein S6 kinase (p70S6k), insulin receptor substrate 1 (IRS-1), and insulin receptor (IR) expression (all *p* < 0.05) but not P/T extracellular regulated kinase ½ (ERK1/2), c-jun N-terminal kinases (JNK), p38 mitogen-activated protein kinases (p38), or insulin-like growth factor 1 receptor (IGF-1R). There were differences between WL and AEX in the change in basal Akt P/T (*p* = 0.05), GSK-3β P/T ratio (*p* < 0.01), p70S6k (*p* < 0.001), ERK1/2 (*p* = 0.01) P/T ratio but not p38, JNK, IRS-1, and IGF-1R P/T ratios. There was a difference between WL and AEX in the insulin stimulation changes in GSK3 which increased more after WL than AEX (*p* < 0.05). In the total group, changes in M were associated with changes in basal total GSK-3β and basal total p70Sk as well as insulin stimulation of total p70Sk. Protein signaling in skeletal muscle provides insight as to mechanisms for improvements in insulin sensitivity in aging and obesity.

## 1. Introduction

Aging and obesity are associated with insulin resistance and interventions such as hypocaloric diet-induced weight loss (WL), aerobic exercise training (AEX), or a combination of the two can improve insulin sensitivity in older, overweight adults [1,2,3,4]. Increased insulin sensitivity stemming from our weight loss and exercise interventions are related to reductions in circulating cytokines independent of decreases in total body and visceral fat [5]; however, mechanisms for improvements in insulin sensitivity could include the ability of insulin to alter the state of certain proteins involved in downstream insulin signaling in skeletal muscle.

Insulin stimulation of skeletal muscle Akt is reduced in insulin resistant states [6,7]. Insulin also promotes phosphorylation of IRS-1 on Ser^307^ in human skeletal muscle during a hyperinsulinemic-euglycemic clamp [8] suggesting that chronic hyperinsulinemia further induces insulin resistance. Glycogen synthase kinase-3 (GSK-3), which phosphorylates and inactivates glycogen synthase, is elevated in insulin resistant states including individuals with type 2 diabetes independent of obesity [9] and in animal models [10]. Mitogen-activated protein kinases (MAPKs) include three main subtypes including extracellular signal-regulated kinase (ERK), c-Jun N-terminal kinase (JNK), and p38 kinase (p38K), which regulate the gene transcription of several messengers involved in survival, apoptosis, cell differentiation, and cardiac remodeling [11].

Few studies have examined the effects of weight loss or exercise on skeletal muscle Akt, GSK-3, MAPKs activation, or other insulin signaling pathways in older, overweight, and obese adults. A two-week diet with weight loss of ~4 kg improved insulin sensitivity in a small group of obese women and men but did not change basal phospho-Akt (pSer473 and Thr308); however, insulin-stimulated biopsies were not obtained [12]. In our previous studies, basal and insulin-stimulated Akt protein expression did not significantly change after a combined WL and exercise program [4] or exercise only [13]. Acute cycle exercise [14] and injury producing or muscle damaging exercise increases JNK activity in human skeletal muscle [15,16]. Additionally, increased JNK activity and Ser^307^ phosphorylation of IRS-1 and a reduction in the activity of Akt are found during muscle disuse atrophy induced by rat hindlimb suspension [17]. However, other studies do not support a role for Akt in exercise/contraction induced signaling in human skeletal muscle [18]. Research is necessary to elucidate the effects of lifestyle interventions on these and other downstream intracellular signaling events in older adults.

The purpose of this study was to examine the effects of 6 month trial of AEX and WL on signaling pathways (Akt, GSK-3β, 70S6K, ERK 1/2, JNK, P38K), and the terminal inhibitory action on IRS-1 tyrosine phosphorylation, IGF-1R, and the IR in the basal state and during hyperinsulinemia in middle-aged and older overweight and obese individuals, and whether the changes in expression and signal transduction are associated with changes in peripheral/whole body insulin sensitivity by a hyperinsulinemic-euglycemic clamp.

## 2. Materials and Methods

Adults aged 45–80 years who resided in the Baltimore/Washington areas of Maryland were eligible for study participation if they were overweight or obese (BMI 25–50 kg/m^2^) but otherwise healthy. Women had undergone menopause at least one year prior to participation to be included. Adults were enrolled who were weight stable (<2.0 kg weight change in past year) and sedentary (<20 min of structured physical activity such as walking, jogging, or swimming for 2x/week). Men and women were excluded for the presence of heart disease, diabetes, cancer, anemia, dementia, untreated dyslipidemia, as well as those with other unstable or chronic diseases affecting the liver, lungs, or kidneys. Informed consent was obtained from all subjects involved in the study. All methods and procedures were approved by the Institutional Review Board at University of Maryland and the VA Research and Development Committee. Potential participants were screened first over the telephone and then in person. They underwent a physical examination including a comprehensive past medical history, fasting blood profile, and a graded exercise treadmill test prior to study participation.

All subjects received instruction in maintaining a weight-stable, Therapeutic Lifestyle Changes (TLC) diet [19] (consuming < 30% of total calories as total fat, 10% as saturated fat, 300 mg of cholesterol, and 2400 mg of sodium per day), by a Registered Dietitian (RD). Subjects were weight stable ±2% on the TLC diet for at least two weeks prior to initial testing. To maintain subject number in group weight loss classes and exercise, subjects were randomly recruited and assigned to group intervention of WL or AEX after instruction in the TLC diet and baseline testing. Subjects in the AEX group had all metabolic tests performed 24–36 h after the last exercise session.

### 2.1. Interventions

Participants in the WL group attended weekly weight loss classes for six months led by a RD for instruction in the principles of hypocaloric diet (−500 kcal/d) according to TLC guidelines. Class topics refreshed participants’ knowledge and included the following: Coping with Slips and Binges, Problem Solving, Healthy Habits, Self-Talk, and Stress Management. Weight loss instructions included following the “heart healthy” dietary modification guidelines plus beginning a 250–500 kcal/d hypocaloric diet with a goal ~5–10% weight loss over the study duration. Participants were instructed by the RD on how to complete a food record. Each person recorded all food eaten, and the estimated quantity of each food. Participant’s weight loss was monitored weekly by the RD and the diets were evaluated by 7-day food records using the American Diabetes Association exchange list system. Those participants in the AEX group met three times per week for six months at our VA annex exercise facility with an exercise physiologist who monitored the subject’s heart rate throughout the exercise session. The exercise program consisted of walking/jogging on a motorized treadmill which began at week one at an intensity of ~50–60% of heart rate reserve (HRR) for 30–40 min. At week 2, it progressed to 55–65% HRR for 45–50 min, week 3 at 60–65% HRR for 50 min, and week 4 at 65–75% HRR. Participants exercised for 50 min at an intensity of ~70–80% of HRR by week 5–8 which was maintained for the remainder of the intervention. Each exercise session included a 5- to 10-min calisthenics and stretching exercise and 5 min of progressive aerobic activity warmup phase and a 5- to 10-min cooldown phase of slow walking after the training period. Heart rate was monitored during each exercise training session using heart rate monitors (Polar Electro Inc., Lake Success, NY, USA).

### 2.2. VO_2_max

VO_2_max was measured by indirect calorimetry (Quark, Cosmed USA, Chicago, IL, USA) during a maximal graded treadmill test [20]. A continuous treadmill test protocol was used in which speed was kept constant but varied for each individual depending on his/her level of conditioning (~3–4 mph). The grade was increased from 0% to 4% at 2 min and was then increased 2% every minute after 4 min until the participant was unable to continue. Expired air was continuously collected to determine the fractional concentrations of oxygen and carbon dioxide. VO_2_max was accepted as valid if two of the three following criteria were met: respiratory exchange ratio ≥ 1.10, maximum heart rate > 90% of age-predicted maximum (220-age), or a plateau in VO_2_ (<200 mL/min change). If such criteria were met, the highest level of VO_2_ was defined as VO_2_max.

### 2.3. Body Composition

Height (cm), weight (kg), and waist and hip circumferences were measured using standardized protocols and used to calculate BMI and WHR. Percent body fat, fat mass, lean body mass, and fat-free mass were determined by dual-energy X-ray absorptiometry (iDXA, LUNAR Radiation Corp., Madison, WI, USA). A single computed tomography (Siemens Somatom Sensation 64 Scanner, Fairfield, CT, USA) scan at L_4_–L_5_ region was used to determine visceral and subcutaneous abdominal adipose tissue area, and analyzed using Medical Image Processing, Analysis, and Visualization, version 7.0.0 (NIH Center for Information Technology, Bethesda, MD, USA). Visceral fat tissue was determined by tracing along the fascial plane defining the internal abdominal wall to distinguish between subcutaneous abdominal fat. A second scan of the right mid-thigh was used to quantify intramuscular fat area (low-density lean tissue), muscle attenuation, subcutaneous fat, and muscle area [3]. Two participants were missing the DXA scan in the WL group and one in the AEX group. There were 5 and 3 missing CT scans in the WL and AEX groups, respectively due to time conflicts or equipment malfunctioning.

### 2.4. Oral Glucose Tolerance Test (OGTT)

After a 12-h overnight fast, the subjects had a blood draw before and at 30-min intervals for 2 h after ingestion of 75 g glucose. Samples were collected in heparinized syringes, placed in prechilled test tubes containing 1.5 mg EDTA/mL blood, centrifuged at 4 °C and stored at −80 °C until analysis. Plasma glucose concentrations were measured using the glucose oxidase method (2300 STAT Plus, YSI, Yellow Springs, OH, USA). Plasma insulin was measured in duplicate by radioimmunoassay (RIA) (Millipore, St. Charles, MO, USA). There was one missing OGTT in the WL group and two missing in the AEX group.

### 2.5. Glucose Clamp and Skeletal Muscle Biopsies

All subjects were weight stabilized (±2%) for at least two weeks prior to metabolic testing including the OGTT and glucose clamp before and after the interventions and were provided all meals as a eucaloric diet for two days before the clamp by a registered dietitian to control nutrient intake [20]. All testing was performed in the morning after a 12-hr overnight fast. Whole body insulin sensitivity was measured using a three-hour hyperinsulinemic-euglycemic clamp technique [21]. Arterialized blood was obtained from a dorsal heated hand vein [22]. Blood samples were obtained every 5 and 10 min for the determination of plasma glucose and insulin levels. A 10 min priming and continuous infusion of insulin (80 mU·m^−2^·min^−1^ Humulin, Eli Lilly Co., Indianapolis, IN, USA) was performed for 180 min with a continuous infusion of 20% glucose solution starting at 10 min. Blood samples were collected in heparinized syringes, placed in prechilled test tubes containing 1.5 mg EDTA/mL of blood and centrifuged at 4 °C for plasma glucose and stored at −80 °C until analysis for plasma insulin. Prior to the start of the clamp and 120-min during the glucose clamp, a vastus lateralis muscle biopsy was taken from each subject under local anesthesia for the measurement of protein expression. Muscle samples were frozen immediately in clamps cooled in liquid nitrogen and stored at −80 °C until assay.

### 2.6. Muscle MSD Electrochemiluminescence (ECL) Protein Assay

Skeletal muscle samples were lyophilized for 48 h and then micro-dissected to be free of obvious connective tissue, fat, and blood. Micro-dissected muscle (~5 mg) was homogenized in lysis buffer (MESO SCALE DIAGNOSTICS, LLC., Gaithersburg, MD, USA. Cat# R60TX-3) containing: 150 mM NaCl, 20 mM Tris pH 7.5, 1 mM EDTA, 1 mM EGTA, 1% Triton X-100 plus Inhibitor Pack (Phosphatase Inhibitor and protease inhibitor cocktail, (MESO SCALE DIAGNOSTICS, LLC., Gaithersburg, MD, USA, Cat# R70AA-1). After shaking on ice for 30 min, the lysate was centrifuged at 14,000× *g* rpm at 4 °C for 10 min (Bachman ALLEGRA X-22R), and the supernatant (muscle lysate) was collected and aliquoted. The muscle lysate was stored in −80 °C until the protein assay. The multiplex assays from MESO SCALE DIAGNOSTICS, LLC., Gaithersburg, MD, USA were used to measure the protein levels in skeletal muscle. The Phospho-(pERK ½ Thr/Tyr:202/204 and 185/187, p38 Thr183/Tyr185 and pJNK Thr180/Tyr182) and Total (ERK 1/2, p38 and JNK) MAPK signaling panel (Cat# K15101D and K15157D), the Phospho-(pAkT Ser473, p70S6K Thr421/Ser424, pGSK-3β Ser9) and Total Akt (Akt, p70S6K, GSK-3β) signaling panel (Cat# K15115D and K15133D) and Phospho- (pIRS-1 Tyr612, pIR Tyr1150/1151, pIGF-1R Tyr1316) and Total (IRS-1, IR, IGF-1R) insulin signaling panel (Cat# K15151C and K15152C) were performed using the MSD SECTOR Imager 2400 according to the manufacturer’s protocol (MESO SCALE DIAGNOSTICS, LLC., Gaithersburg, MD, USA). The same amount of total protein (40 μg in 25 μL volume) per well was loaded in each assay. The same lysis buffer was used for diluting samples. Our pooled human skeletal muscle lysate served as control and was loaded on each assay plate. The data for the ratio of Phospho-/Total (P/T) was calculated. It should be noted that some of the ratios for Akt panel are greater than 100% because the phosphorylated protein and total protein were analyzed with different kits and the P/T*100 ratios serve as the ratio (AU) of the signals.

### 2.7. Statistical Analyses

For the hyperinsulinemic-euglycemic clamps, M was calculated from the amount of glucose infused after correction for glucose equivalent space (glucose space correction). Baseline comparisons of the two intervention groups were performed using unpaired Student’s *t*-tests. Paired Student’s *t*-tests were used to compare muscle expression within each of the groups including (1) basal vs. insulin-stimulated pre-intervention; (2) basal vs. insulin-stimulated post intervention; and (3) basal preintervention vs. basal postintervention. One-way ANOVA was used to test differences in changes in outcomes between interventions. Paired *t*-tests tested the effects for the intervention within groups. The Shapiro–Wilk test of normality was used to test for normal distribution of data. Pearson correlations were used to assess relationships between key variables. Statistical significance was set at a two-tailed *p* < 0.05. Data were analyzed using SPSS (IBM SPSS Statistics 27, Armonk, NY, USA); results are expressed as mean ± SEM.

## 3. Results

Participants (*n* = 217) who expressed initial interest in the study were screened for eligibility. 119 were consented, enrolled and underwent a physical history and exam and 27 failed screening. Twenty-four withdrew during the TLC period or during baseline testing and were not randomized-mostly due to personal reasons (family/self) and lack of time. Thirty-eight were assigned to WL (16 dropped during WL); thirty were assigned to AEX (10 dropped during AEX). Participants who completed the interventions (*n* = 3 per group) that were outside the age range of 50–70 were not included in this paper, although the inclusion would not have changed the results. Thus, participants were 50–70 years of age, 71% Caucasian, 29% African American and completed the WL (*n* = 18, *n* = 10 males, *n* = 8 females) or AEX (*n* = 17, *n* = 9 males, *n* = 8 females). Age and BMI were not different between males and females but men had higher waist, WHR and FFM (*p* < 0.001), and women higher %fat (*p* < 0.001).

### 3.1. Body Composition and Fitness

Subject characteristics before and after six months of either WL or AEX are reported in Table 1. At baseline, age, body weight, BMI, and VO_2_max were not different between WL and AEX. In addition, waist circumference, hip circumference, WHR, fat mass, fat-free mass, visceral fat area, subcutaneous abdominal fat area, sagittal diameter, mid-thigh muscle area, mid-thigh subcutaneous fat area, mid-thigh intramuscular fat area, and muscle attenuation were not significantly different between treatment arms (WL and AEX) at baseline. There were between group differences in the change in body weight, VO_2_max, fat mass and FFM (all *p* < 0.001) as well as percent body fat, subcutaneous abdominal fat, and intramuscular fat (*p* < 0.05) but not visceral fat or muscle area. Body weight and BMI decreased after WL (*p* < 0.0001) accompanied by reductions in waist circumference, hip circumference, percent body fat and fat mass (*p* < 0.0001) and WHR (*p* < 0.05). Lean body mass and fat-free mass all decreased after WL (*p* < 0.01). Visceral fat (*p* < 0.05) and subcutaneous abdominal fat (*p* < 0.01) both decreased ~16%, along with a decrease in mid-thigh subcutaneous fat (*p* < 0.05) but mid-thigh muscle area, intramuscular fat, and muscle attenuation did not significantly change with WL. In the AEX group, percent body fat decreased (*p* < 0.01), and fat-free mass increased (*p* = 0.05). Body weight, BMI, hip circumference, WHR, lean body mass, and FFM did not change with AEX but waist circumference and percent body fat decreased (*p* < 0.05). There were no significant changes in visceral fat, subcutaneous abdominal fat, or sagittal diameter after AEX. Intramuscular fat decreased after AEX (*p* = 0.01). There were no significant changes in mid-thigh muscle area, subcutaneous fat, and muscle attenuation after AEX.

### 3.2. Glucose Metabolism

There were between group differences in the change in fasting glucose (*p* < 0.05), GlucAUC_120_ (*p* = 0.005), GlucAUC_180_ (*p* < 0.05), but not changes in fasting insulin, InsulinAUC_120_, InsulinAUC_180_, and M. Fasting glucose (*p* < 0.001), glucose_120 min_ of the OGTT (*p* < 0.01), glucose_180 min_ (*p* < 0.05), fasting insulin (*p* = 0.005), insulin_120 min_ (*p* = 0.01), and insulin_180 min_ (*p* < 0.01) decreased after WL. In addition, there were significant reductions in GlucAUC_120_ (*p* < 0.001), GlucAUC_180_ (*p* < 0.001), InsulinAUC_120_ (*p* = 0.01), and InsulinAUC_180_ (*p* < 0.05) with WL. InsulinAUC_180_ decreased after AEX (*p* < 0.05) but there were no significant changes in glucose levels, fasting insulin, or InsulinAUC_120_ after AEX. Insulin sensitivity (M) increased after WL (umol·kg^−1^·min^−1^, *p* < 0.001 and umol·kg_FFM_^−1^·min^−1^, *p* < 0.005) and AEX (μmol·kg^−1^·min^−1^, *p* = 0.005 and umol·kg_FFM_^−1^·min^−1^, *p* = 0.01).

### 3.3. Effects of Insulin Stimulation on Skeletal Muscle

#### 3.3.1. Akt Signaling Panel

**Figure 1:**
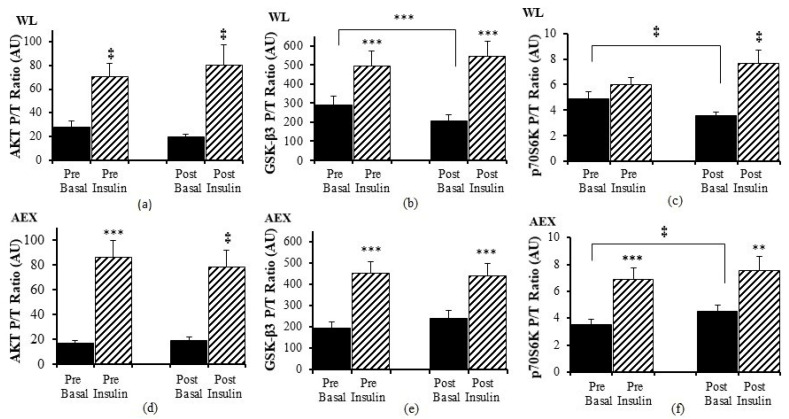
Skeletal muscle Akt, GSK-3β and p70S6K phosphorylation, and total protein expression ratio during basal (solid) and insulin-stimulated (striped) pre and post WL (**a**–**c**) and AEX (**d**–**f**). Paired *t*-tests between pre basal vs. pre insulin, post basal vs. post insulin, and pre vs. post basal analyses within WL and within AEX were performed using the SPSS software version 27 (SPSS Inc., Chicago, IL, USA). ** *p* ≤ 0.01; *** *p* ≤ 0.001, ‡ *p* ≤ 0.005.

**Figure 2:**
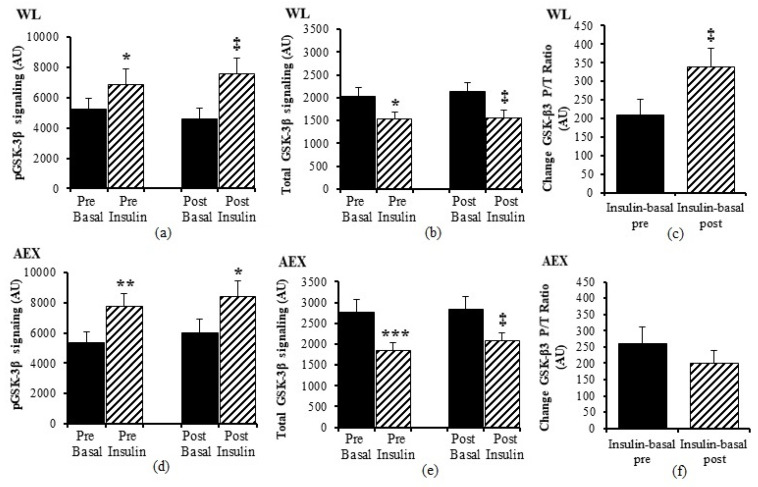
Skeletal muscle GSK-3β phosphorylation and total protein expression level at basal (solid) and insulin-stimulated (striped) pre and post of WL (**a**,**b**) and AEX (**d**,**e**). Change GSK3ß (Insulin-basal) at pre and post of WL (**c**) and AEC (**f**). Paired *t*-tests between pre basal vs. pre insulin, post basal vs. post insulin, and pre vs. post basal analyses within WL and within AEX were performed using the SPSS software version 27 (SPSS Inc., Chicago, IL, USA). (* *p* < 0.05; ** *p* < 0.01; *** *p* < 0.001, ‡ *p* < 0.005.

We examined the effects of the insulin infusion during the glucose clamp on the proteins before as well as after the interventions (e.g., basal pre vs. insulin stimulated pre and basal post vs. insulin stimulated post). In the WL group, insulin stimulation in skeletal muscle activated both Akt (*p* < 0.005) and GSK-3β P/T ratio (*p* < 0.001) pre and post WL (Figure 1a,b). Insulin stimulation did not change p70S6k P/T ratio pre WL and increased post WL (*p* < 0.005, Figure 1c). In the AEX group, insulin stimulation also activated skeletal muscle Akt P/T ratio pre (*p* < 0.001) and post (*p* < 0.005) AEX (Figure 1d). Likewise, skeletal muscle GSK-3β P/T increased with insulin stimulation both pre and post AEX (all *p* < 0.001, Figure 1e). Insulin activated p70S6k P/T pre and post (*p* < 0.001) AEX (Figure 1f). Total protein expression of Akt and p70S6k did not change with insulin in vivo either before or after either intervention (data not shown). In both groups, total protein expression of GSK-3β decreased with insulin pre WL (*p* < 0.05) and AEX (*p* < 0.001) and post WL and AEX (both *p* < 0.005) (Figure 2b,e). Phosphorylation protein expression of Akt, GSK-3β and p70Sk increased with insulin pre WL (all *p* < 0.05) and pre AEX (all *p* < 0.01). After the interventions, phosphorylation protein expression increased for Akt in both groups (both *p* < 0.05), for GSK-3β (WL: *p* < 0.005 and AEX: *p* < 0.05), and for p70S6k (WL: *p* = 0.001 and AEX: *p* < 0.01). P/T ratio of basal GSK-3β (*p* < 0.001) and p70S6K (*p* < 0.005) decreased after WL (Figure 1b,c). P70S6k increased after AEX (*p* < 0.005) (Figure 1f).

#### 3.3.2. MAPK Signaling Panel

**Figure 3:**
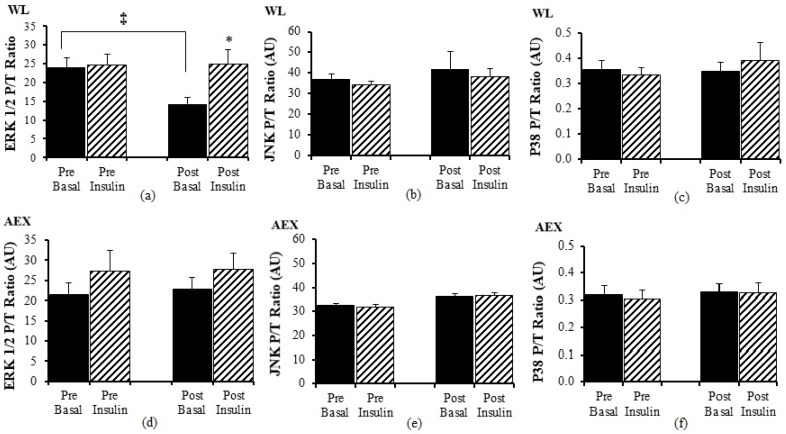
Skeletal muscle ERK1/2, JNK and p38 phosphorylation, and total protein expression ratio during basal (solid) and insulin-stimulated (striped) pre and post WL (**a**–**c**) and AEX (**d**–**f**). Paired *t*-tests between pre basal vs. pre insulin, post basal vs. post insulin, and pre vs. post basal analyses within WL and within AEX were performed using the SPSS software version 27 (SPSS Inc., Chicago, IL, USA). * *p* ≤ 0.05; ‡ *p* ≤ 0.005.

There was no significant change in ERK1/2 P/T ratio with insulin pre WL but post WL, there was an increase in ERK 1/2 P/T ratio (*p* < 0.05 Figure 3a) during insulin stimulation. Ratio of basal P/T ERK 1/2 decreased after WL (*p* < 0.05) (Figure 3a). Insulin stimulation did not change JNK P/T ratio pre or post WL or AEX (Figure 3b,e). There was no significant effect of insulin for ERK 1/2 P/T ratio (Figure 3d) pre or post AEX. There was no change (basal vs. insulin stimulated) in P38 P/T ratio pre or post WL (Figure 3c) or AEX (Figure 3f). Total protein expression of ERK 1/2, JNK, and P38 did not change with insulin in vivo either before or after either intervention except for P38 post AEX (*p* < 0.05) (data not shown).

#### 3.3.3. Insulin Signaling Panel

**Figure 4:**
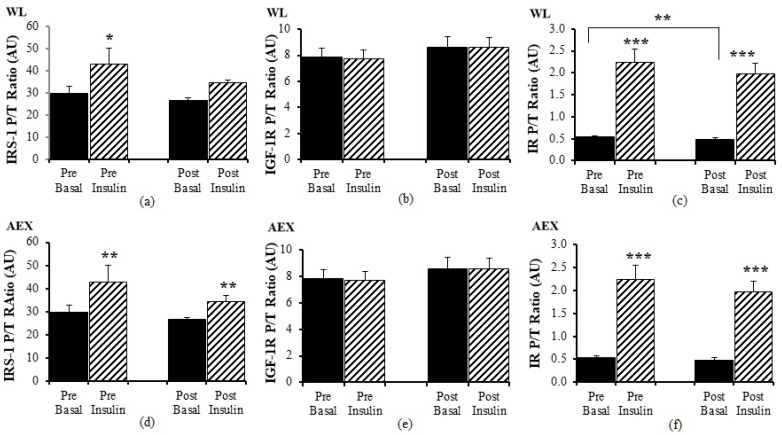
Skeletal muscle IRS-1, IGF-1R and IR phosphorylation, and total protein expression ratio during basal (solid) and insulin-stimulated (striped) pre and post WL (**a**–**c**) and AEX (**d**–**f**). Paired *t*-tests between pre basal vs. pre insulin, post basal vs. post insulin, and pre vs. post basal analyses within WL and within AEX were performed using the SPSS software version 27 (SPSS Inc., Chicago, IL, USA). * *p* ≤ 0.05; ** *p* ≤ 0.01; *** *p* ≤ 0.001.

Pre WL, insulin activated IRS-1 P/T ratio (*p* < 0.05) but there was no change with insulin stimulation during the clamp post WL (Figure 4a). There was no significant effect of insulin stimulation on IGF-1R P/T ratio pre and post WL (Figure 4b) or pre and post AEX (Figure 4e). In the AEX group, insulin activated IRS-1 P/T ratio pre and post (*p* = 0.01, Figure 4d) AEX. Insulin activated IR P/T ratio pre (*p* < 0.001) and post (*p* < 0.001) WL (Figure 4c) and AEX (Figure 4f). Basal P/T ratio of IR decreased after WL (*p* < 0.01) (Figure 4c). Total protein expression of IRS-1, IGF-1R, and IR did not change with insulin in vivo either before or after either intervention (data not shown).

### 3.4. Effects of the Interventions on Skeletal Muscle Expression of Signaling Proteins

We examined changes in P/T protein expression in the basal state between and within groups. There were differences between WL and AEX in the change in basal Akt P/T (*p* = 0.05), GSK-3β P/T ratio (*p* < 0.01), p70S6k (*p* < 0.001), ERK1/2 (*p* = 0.01) P/T ratio but not p38, JNK, IRS-1, and IGF-1R P/T ratios. Within groups, WL decreased P/T ratio of basal GSK-3β (*p* < 0.001), p70S6k (*p* = 0.005), ERK1/2 (*p* = 0.002), IR (*p* = 0.01), but did not change basal Akt, JNK, P38, IRS-1, and IGF-1R protein expression and phosphorylation. AEX increased basal p70S6k P/T ratio (*p* < 0.05). Basal Akt, GSK- 3β, ERK1/2, JNK, P38, IRS-1, IGF-1R and IR P/T protein expression did not change with AEX.

We next examined changes in P/T protein expression in the magnitude with insulin stimulation between groups. There were no significant differences in P/T changes with insulin stimulation between groups [(insulin post–basal post)-(insulin pre–basal pre)] in Akt, p70S6k, ERK1/2, JNK, p38, IRS, IGF-1R, and IR. There was a difference between WL and AEX in the insulin stimulation changes in GSK3 which increased more after WL than AEX (*p* < 0.05). Within each group, there were no within group changes in the effect of insulin before and after the interventions (insulin-basal pre vs. insulin-basal post) in Akt, ERK1/2, JNK, p38, IRS-1, IGF-1R, and IR in either group. In the WL group, insulin-stimulated increase in GSK-3β (*p* < 0.01) and p70S6k (*p* < 0.05) P/T phosphorylation was greater after the intervention. Changes in insulin stimulation in GSK-3β and p70S6k P/T protein expression did not change significantly after AEX.

Basal total protein for any of the proteins was not different between WL and AEX groups before or after the interventions. There was also no significant changes in basal total protein levels for Akt, GSK-3β, p70S6k, ERK1/2, JNK, p38, IRS-1, IGF-1R, or IR in either group.

### 3.5. Relationships of Muscle Signaling and Insulin Sensitivity

Changes in the proteins within pathways were related to each other in the total group. The change in basal Akt P/T expression was associated with changes in basal GSK-3β P/T (r = 0.48, *p* < 0.005), p70S6k (r = 0.34, *p* < 0.05), and IR (r = 0.68, *p* < 0.001). Changes in basal GSK-3β P/T expression were associated with changes in p70S6k P/T (r = 0.56, *p* < 0.001) and ERK 1/2 P/T expression (r = 0.57, *p* < 0.001). Changes in basal p70S6k were associated with ERK 1/2 (r = 0.66, *p* < 0.001) and change in JNK P/T expression with the change in p38 P/T expression (r = 0.73, *p* < 0.001). In the total group, changes in M were associated with changes with basal total GSK-3β (r = 0.37, *p* < 0.05) and basal total p70Sk (r = 0.38, *p* < 0.05) as well as insulin stimulation of total p70Sk (r = −0.40, *p* < 0.05) and tended to be with total GSK-3β (r = −0.34, *p* = 0.06). In the total group, changes in body weight were associated with changes in basal p70S6k P/T expression (r = 0.46, *p* < 0.01) and tended to be associated with changes in basal GSK-3β P/T expression (r = 0.30, *p* = 0.06). In the total group, the change in visceral fat and intramuscular fat were not associated with the change in M. However, the change in fat mass was associated with an increase in M in μmol·kg^−1^·min^−1^ (r = −0.41, *p* < 0.05).

## 4. Discussion

Our results indicate that a six-month intervention of either caloric restriction WL or supervised moderate to high intensity AEX training improves insulin sensitivity. Reductions in body weight and total body fat mass were greater in the WL group as expected, whereas AEX increased VO_2_max, muscle mass, and reduced intramuscular fat. WL improved glucose levels during the OGTT whereas AEX did not. Our results also indicate that insulin stimulation has a robust effect to increase the protein expression in several pathways including P/T ratio of Akt, GSK-3β, p70S6k, IRS-1, and IR in overweight and obese middle-aged and older adults. When we examined the effects of the interventions on these signaling pathways, we found that weight loss reduces the basal P/T expression and the change with insulin stimulation of several proteins including GSK-3β and p70S6k whereas AEX increases p70S6k. The increase in insulin sensitivity with WL and AEX is related to changes in both basal levels and insulin stimulation of total protein for GSK-3β and p70S6k.

Insulin plays an important role to facilitate the transmembrane transport of glucose into certain tissues. We studied the downstream insulin molecular signaling pathways to elucidate the role of insulin infusion during euglycemia and the effects of two different lifestyle interventions aimed to improve insulin sensitivity. Insulin actives glycogen synthase through insulin-induced inhibition of GSK3. The inhibition of GSK3 is through the stimulation of Akt and p70S6K phosphorylation, which in turn stimulates the serine phosphorylation of GSK3β at Ser9 [23,24,25,26]. Our study shows that insulin increases the phosphorylation of Akt by 4-fold, GSK3β by 1.5- to 2-fold, and p70S6k by 1 to 2-fold in skeletal muscle. In animal models, exercise increased p70S6k (Thr 389) and Akt (Thr 308) phosphorylation immediately after and during recovery in rats [27]. Another report showed that exercise induced greater p70S6KThr412 in insulin-stimulated muscles, regardless of diet in the rat model [28]. Compared with the muscle from lean and weight-matched obese nondiabetic individuals, in diabetic muscles, GSK-3α/β protein levels and enzyme activities were increased about ~64% and ~286%, respectively, and negatively correlated with glycogen synthase activity and insulin-induced glucose utilization [9]. Chronic treatment with GSK-3 inhibitors leads to down-regulation of insulin receptor (IR), IRS-1, IRS-2, and Akt levels [29]. Our results show the basal p70S6k phosphorylation increased after AEX. In contrast, basal GSK3β and p70S6k phosphorylation decreased after WL. The insulin stimulation of Akt, GSK3β and p70Sk phosphorylation level did not change after WL and AEX and yet, the two interventions improved insulin sensitivity. Moreover, the reasons for the different effects by intervention on basal p70S6k and GSK3β are not clear. Some studies have examined changes in the basal expression of various proteins, but less so with hyperinsulinemia. In a small (*n* = 6) study of middle-aged adults, there was a nonsignificant increase in basal skeletal muscle phosphorylation of GSK3β protein expression after a 7% decrease in BMI and the greater the increase, the smaller the improvement in glucose utilization [30]. It has been shown that GSK3β phosphorylation is greater in older adults than young individuals but total protein content does not differ [31] and that a short exercise duration of 12 weeks of aerobic or resistive training has no effect on insulin-induced phosphorylation of GSK-3β [31]. Our data indicate that in vivo insulin stimulation has the opposite effect on the phosphorylation and total protein of GSK3ß (e.g., significantly increases skeletal muscle GSK3ß phosphorylation and decreases total protein level). This is true both before and after each intervention. Therefore, insulin might have dual actions on GSK3β: 1) reduce GSK3β protein activity by decreasing total GSK3β protein level; and 2) increase the inactivated GSK3β by increasing the GSK3β phosphorylation. These two actions can decrease the amount of active of GSK3β and promote glycogen synthesis. Furthermore, we found that the change in GSK3β P/T ratio due to insulin significantly increased after WL indicating that the phosphorylation of GSK3β protein became more sensitive to insulin after WL. Yet, this was not observed after AEX, indicating different responses by these behavioral interventions. GSK3 inhibitors are used in the treatment of diabetes due to the down regulation of IR, IRS-1, and Akt levels [29]. Our data show that the change of GSK3β P/T ratio (insulin-basal) was increased, basal IR P/T ratio was decreased, and insulin sensitivity or M improved after a six-month WL program, perhaps indicating that skeletal muscle requires less IR when more insulin sensitive.

The role of p38MAPK in insulin resistance is complex. In obesity, it contributes to the development of insulin resistance [32]. In high-fat diet fed mice, inhibition of p38 MAPK reduces insulin resistance and prevents obesity [32]. Furthermore, p38 negatively regulates insulin-stimulated glucose transport activities [32]. It also has been shown that inhibiting p38MAPK can partially restore impaired insulin-stimulated glucose transport activity in rat skeletal muscle [33]. Basal phosphorylation of skeletal muscle p38 MAPK is higher in adults with type 2 diabetes than controls [34] but phosphorylation of another MAPK, ERK1/2 is normal [35]. Although a different type of insulin stimulus, in vivo insulin does not change p38 MAPK phosphorylation in adults with type 2 diabetes [34]. We not only did not see any significant change in p38 MAPK phosphorylation after insulin stimulation, but also did not observe a change in the basal expression after the interventions. One study showed that insulin could increase p38 MAPK phosphorylation in skeletal muscle of non-diabetic subjects, but not in Type 2 diabetic patients [32]. It could be that the lack of change of in the P/T p38 MAPK in our study is due to obesity associated insulin resistance in our study population.

It is well-known that the insulin receptor substrate-1 (IRS1) and IR are critical elements in insulin-signaling pathways. Activated p38 MAPK inhibits IRS1 of insulin signaling through inhibitory phosphorylation [32]. Increasing p38MAPK activation is associated with glucose intolerance and hyperinsulinemia in high fat fed mice and obese mice [36]. Moreover, p38MAPKα overexpression enhances IRS-1 serine phosphorylation and decreases insulin-induced IRS-1 tyrosine phosphorylation [36]. We found a ~1.5 fold increase in IRS-1 with insulin stimulation before and after AEX with a smaller effect in the WL group. Likewise, there was a robust increase (>three-fold in AEX and two-fold in WL) in IR expression with insulin activation. Thus, defects in insulin signaling network in obesity are difficult to modify, as suggested by our findings of changes only in the IR after either WL or AEX on the MAPK and insulin signaling pathways.

During exercise, p38 facilitates glucose transport in an insulin-independent manner and improves insulin-dependent glucose transport through glucose transporters, Glut1 and Glut4 [32]. Acute resistive and aerobic exercise significantly increases ERK1/2 phosphorylation and IGF-1R [37,38]. Our data shows that there were no significant changes in ERK1/2 and IGF-1R phosphorylation after 6-month AEX training, perhaps suggesting that acute vs. chronic exercise has different effects on these proteins as ERK1/2 phosphorylation decreased and IGF-1R did not change after WL. Our findings that there were no relationships between changes in M and these nine proteins indicate that they do not explain the improvements in insulin sensitivity with the WL and AEX.

Our data shows that changes in basal protein levels correlate with one other. Furthermore, changes in insulin sensitivity are associated with the changes of insulin stimulation of total p70S6k in the total group. We speculate that insulin promotes muscle glycogen synthesis by means of interaction with its receptor on the cell surface, which then activates Akt and p70S6K. The activated Akt and p70S6K further phosphorylate GSK3β at ser9 and reduces its activity, which promotes the activation of glycogen synthase (GS), thus increasing the muscle glycogen.

In addition to muscle mechanisms for insulin resistance, central body fat (visceral fat and subcutaneous abdominal fat) is associated with insulin resistance [20,39]. Others report that intermuscular fat directly regulates skeletal muscle insulin sensitivity [40], so we would have expected to see a relationship between reduction in IMAT and improvements in glucose utilization.

Our study limitations include the lack of ability to test for sex differences and generalizability to populations with chronic disease. Although our study was sufficiently powered to detect differences in the primary muscle outcomes, it was slightly underpowered to detect differences between exercise and weight loss effects on the secondary outcome, glucose utilization. Our study has several strengths including the conduct of hyperinsulinemic-euglycemic clamps with basal and insulin-stimulated muscle biopsies, control of dietary intake prior to metabolic testing, assessment of both phosphorylated and total levels of each protein, the structured and rigorous weight loss classes and exercise training, and the long duration of each intervention.

## 5. Conclusions

A six-month weight loss program or aerobic training intervention improve insulin sensitivity in older overweight and obese adults. Aerobic exercise improves VO_2_max, increases muscle mass and reduces intramuscular fat. Weight loss reduces body weight, body fat, and improves glucose tolerance. In vivo insulin stimulation increases the expression of Akt, GSK-3β, p70S6k, IRS-1, and IR in overweight and obese middle-aged and older adults and changes in basal total GSK-3β and basal total p70Sk as well as insulin stimulation of total p70Sk are related to improvements in insulin sensitivity. Further study of the mechanisms of action in skeletal muscle that contribute to improvements in glucose metabolism in this study group would provide insight into the adaptations of skeletal muscles to exercise and weight reduction in aging and obesity.

## Figures and Tables

**Table 1:** Clinical and metabolic characteristics.

	Weight Loss (*n* = 18)		Aerobic Exercise Training (*n* = 17)	
	Pre	Post	Pre	Post
Age (years)	60.2 ± 1.3		60.5 ± 1.1	
Weight (kg)	102.4 ± 4.1	93.0 ± 3.6 ‡,a	94.3 ± 3.9	93.2 ± 4.6
BMI (kg/m^2^)	35 ± 1	33 ± 1 ‡	33 ± 1	33 ± 1
Waist circumference (cm)	110.7 ± 2.9	103.9 ± 2.6 ‡	102.7 ± 3.1	100.9 ± 3.5 *
Hip circumference (cm)	119.5 ± 2.2	113.9 ± 2.1 ‡	116.4 ± 2.7	115.8 ± 2.7
WHR	0.94 ± 0.02	0.91 ± 0.02 *	0.88 ± 0.03	0.87 ± 0.03
VO_2_max (mL/kg/min)	23.72 ± 1.24	25.52 ± 1.39 ‡	25.11 ± 1.08	29.30 ±1.45 ‡a
VO_2_max (L/min)	2.40 ± 0.14	2.36 ± 0.14	2.40 ± 0.14	2.72 ± 0.14 ‡a
**Body Composition**				
Percent body fat	43.2 ± 1.8	39.7 ± 2.0 ‡ a	39.6 ± 2.0	38.3 ± 2.2 †
Fat mass (kg)	44.1 ± 2.3	37.4 ± 2.2 ‡ a	38.0 ± 2.7	36.8 ± 3.1
Lean body mass (kg)	55.8 ± 3.2	54.5 ± 3.1 †	54.6 ± 2.6	55.5 ± 2.9
Fat-free mass (kg)	58.1 ± 3.2	56.8 ± 3.1 †a	57.4 ± 2.7	58.4 ± 3.0 *
Visceral fat area (cm^2^)	205.7 ± 22.9	173.0 ± 14.0 *	172.0 ± 20.7	146.7 ± 19.6 b
Subcutaneous abdominal fat area (cm^2^)	464.1 ± 33.5	389.7 ± 36.4 *	395.5 ± 41.3	388.6 ± 44.5 b
Sagittal diameter (cm)	29.8 ± 0.8	25.4 ± 1.0 *	25.0 ± 1.1	25.6 ± 1.0
Mid-thigh muscle area (cm^2^)	91.4 ± 6.5	91.1 ± 7.1	90.4 ± 7.3	90.1 ± 6.4
Mid-thigh subcutaneous fat area (cm^2^)	137.0 ± 16.1	118.7 ± 17.2 *	138.3 ± 19.9	130.8 ± 19.6
Mid-thigh intramuscular fat area (cm^2^)	35.0 ± 3.1	36.1 ± 2.6	36.2 ± 3.0	33.1 ± 3.3 †
Mid-thigh muscle attenuation (HU)	36.2 ± 1.6	36.5 ± 1.7	35.1 ± 1.8	35.0 ± 2.1
**Glucose Tolerance**				
Fasting glucose (mmol/L)	5.63 ± 0.14	5.19 ± 0.09 ‡ a	5.48 ± 0.70	5.33 ± 0.59
120-min postprandial glucose (mmol/L)	8.38 ± 0.57	7.27 ± 0.54 †	7.38 ± 2.39	7.18 ± 2.38
180-min postprandial glucose (mmol/L)	5.40 ± 0.31	4.94 ± 0.36 *	5.17 ± 0.36	4.90 ± 0.32
Fasting insulin (pmol/L)	113 ± 11	84 ± 9 **	109 ± 11	97 ± 10
120-min insulin (pmol/L)	757 ± 102	521 ± 60 †	603 ± 104	506 ± 61
180-min insulin (pmol/L)	361 ± 61	229 ± 54 †	239 ± 37	211 ± 49
Glucose AUC (mmol/L/120 min)	1054 ± 53	914 ± 50 ‡b	964 ± 216	962 ± 224
Glucose AUC (mmol/L/180 min)	1433 ± 73	1283 ± 70 ‡b	1358 ± 322	1333 ± 317
Insulin AUC (pmol/L/120 min)	72,065 ± 7235	53,386 ± 6674 †	66,335 ± 7686	60,892 ± 7350
Insulin AUC (pmol/L/180 min)	110,109 ± 13,897	79,698 ± 9619 *	92,009 ± 10,434	82,778 ± 9568 *
**Insulin Sensitivity**				
M(µmol/kg/min)	22.54 ± 2.22	31.90 ± 2.47 ‡	25.58 ± 2.69	33.24 ± 3.09 **
M(µmol/kg_FFM_/min)	41.22 ± 4.86	53.50 ± 4.66 **	46.53 ± 4.84	54.36 ± 5.38 †

Baseline comparisons of the two intervention groups were performed using unpaired Student’s *t*-tests. One-way ANOVA was used to test differences in changes in outcomes between interventions (a: *p* < 0.001, b: *p* < 0.05). Paired *t*-test compared each characteristic within groups: * *p* ≤ 0.05, † *p* ≤ 0.01, ** *p* ≤ 0.005, ‡ *p* ≤ 0.001. Analyses were completed with the SPSS software version 27 (SPSS Inc, Chicago, IL, USA). Data shown are mean ± SD.

## Data Availability

Data will be available upon reasonable request.
